# Unlicensed driving among young drivers in North Carolina: a quasi-induced exposure analysis

**DOI:** 10.1186/s40621-022-00391-9

**Published:** 2022-08-16

**Authors:** Yudan Chen Wang, Robert D. Foss, Arthur H. Goodwin

**Affiliations:** 1 North Carolina A&T State University, Proctor Hall 267, 1601 E. Market St., Greensboro, NC 27411 USA; 2 University of North Carolina at Chapel Hill, Chapel Hill, NC USA

**Keywords:** Unlicensed driving, Young drivers, Graduated driver licensing, Quasi-induced exposure

## Abstract

**Background:**

Little is known about the prevalence of driving among teenagers who have not yet obtained a license. The primary objective of the present study was to estimate the prevalence of unlicensed driving among young drivers using the quasi-induced exposure (QIE) approach and to determine whether unlicensed driving was more common among minority and lower-income teenagers. Additionally, we examined whether unlicensed driving among adolescents increased following the implementation of a graduated driver licensing (GDL) system and whether GDL differentially affected minority and low-income adolescents.

**Methods:**

Using North Carolina crash and driver license data, we identified 90,267 two-vehicle crashes from 1991 through 2016 where only one driver was considered contributory and the non-contributory driver was a White or Black 16 or 17 years old. In the QIE approach, these non-contributory young drivers are assumed to be representative of all adolescents driving in the state during this time period. The prevalence of unlicensed driving among adolescents by age and year was estimated by identifying the proportion of non-contributory drivers who had never been licensed by the time of their involvement in these two-vehicle crashes. We further conducted logistic regression analyses to examine the likelihood of a non-contributory young driver being unlicensed as a function of race, neighborhood income level, and licensing era (prior to or after GDL was implemented).

**Results:**

During the 26 years for which data were available, the mean annual prevalence of unlicensed driving was 1.2% for 16-year-olds and 1.7% among 17-year-olds. Young Black drivers and individuals living in lower-income neighborhoods were somewhat more likely to drive before obtaining a license, but the rates of unlicensed driving among these groups were also quite low. Unlicensed driving increased slightly for 17-year-olds following the implementation of GDL, but returned to previous levels after a few years.

**Conclusion:**

Unlicensed driving among adolescents in North Carolina is substantially less common than suggested by previous self-report studies and analyses of fatal crash data.

## Background

The move to graduated driver licensing (GDL) for young beginning drivers in the USA began a quarter century ago. Since then, there has been a dramatic increase in research on young drivers, along with a marked advance in the sophistication of that research. Nonetheless, little is known about the prevalence of driving among teenagers who have not yet obtained a license. This is somewhat puzzling given the concerns that have long been expressed about the possible unintended effects of GDL on unlicensed driving.

Self-report has been the most common approach to studying unlicensed driving (or unlicensed drivers) among adolescents. In a nationally representative school-based survey, 4% of 9th–11th grade students in the U.A reported driving at least one hour a week without a license (Elliott et al., 2008). Additionally, Black and Hispanic teens, and those living in rural areas or city centers, were more likely to report having driven without a license. Heck et al. (2008) conducted a survey of a convenience sample of high-school seniors in California and also found that both driving and unlicensed driving varied widely by race/ethnicity. Black and Latino respondents were only one-third as likely as non-Hispanic Whites to report any driving. But among those who reported driving, Blacks (36%) and Latinos (30%) were far more likely to be unlicensed than non-Hispanic White drivers (3%). This difference appeared to reflect socioeconomic factors more strongly than race/ethnicity per se. Respondents from schools characterized as low income were 33 times more likely to report driving without a license compared to teens from upper-income schools. Analyses of Youth Risk Behavior Survey data in Montana—a more representative sample than the California study—found that 5% of high-school students old enough to obtain a license reported driving “regularly on public roads” prior to licensing (Hanna et al. 2013). The existing literature clearly suggests that unlicensed driving is more common among minority and socioeconomically disadvantaged teenagers. However, because of the different sample coverages, sampling approaches, and operationalizations of unlicensed driving, available survey data provide only a general indication of the extent of unlicensed driving among adolescents.

Analyses of crash data provide another, albeit also problematic, way to estimate unlicensed driving. A few studies have reported the proportion of crashes that involve unlicensed drivers (AAA Foundation for Traffic Safety 2011; Blows et al. 2005; Møller & Janstrup 2021; NHTSA 2014). However, lack of a license is associated with crash risk, so the prevalence of unlicensed drivers on the road in general would be different (lower). This is because the kinds of behavior that result in licenses being revoked or suspended (excessive speeding, reckless driving, drink-driving) also lead to crashes (Sagberg 2018). In addition, most crash analysis studies of unlicensed driving have looked exclusively at the general driving population, rather than adolescent drivers.

In contrast to these typical approaches, the quasi-induced exposure (QIE) approach allows researchers to estimate the relative risk of a certain behavior or outcome while accounting for exposure (Jiang et al. 2014), avoiding the limitations inherent in self-report surveys and the complications in analyzing the licensing status of drivers involved in crashes. The QIE analyses assume that non-contributory drivers involved in two-vehicle crashes provide a reasonably representative sample of the driving population; that is, they are “randomly” selected from the driving population on the road at that time by the contributory drivers. Previous studies have supported the representativeness assumption of the QIE approach (e.g., Jiang & Lyles 2010; Zhao et al. 2019), including for young drivers (Curry et al. 2016). Curry (2017) subsequently used this approach to estimate compliance with night and passenger limits among New Jersey intermediate-license drivers. Estimates derived using QIE were highly similar to previous estimates obtained in naturalistic driving studies. In view of its desirable features, we used the QIE approach in this study to measure unlicensed driving among the entire young driver population in a state, rather than limited samples whose representativeness and measurement validity are sometimes questionable.

We also examined whether GDL may have contributed to the prevalence of unlicensed driving among adolescents. Most US states have adopted GDL systems to reduce the high crash rates of young, inexperienced drivers. Extensive research has documented the benefits of GDL for 16- and 17-year-old novice drivers (Foss et al. 2001; Shope 2007; Shope et al. 2001; Williams 2017; Williams & Shults 2010; Williams et al. 2012). Nonetheless, some have speculated that GDL might contribute to the prevalence of unlicensed driving. The suspicion is that adolescents may choose to drive without a license until they can obtain one without navigating the multi-step GDL process. A related long-standing concern is that this more involved approach to licensing may create a particular burden for socioeconomically disadvantaged teens, who may not have the necessary adult support system, or the economic resources needed, to meet the requirements of GDL. Recently, Vaca et al. (2021) examined this question as part of a more extensive analysis of teen driver licensing and found no evidence that GDL elements contributed to delays in licensing.^1^ Other studies of this matter indicate that economic issues (household income, lack of vehicle access, economic recessions) far outweigh GDL as a reason for delayed licensure among teens (HLDI 2013; Shults & Williams 2013; Tefft & Foss 2019; Tefft et al. 2014). However, no study has yet directly examined whether the adoption of GDL led to an increase in unlicensed driving.

This study addressed the following three questions: (1) What is the prevalence of unlicensed driving among adolescents in North Carolina (NC)? (2) Is unlicensed driving more common among adolescents in minority and low-income families? (3) Did unlicensed driving among adolescents increase following the implementation of a GDL system, and did GDL differentially affect minority and low-income adolescents? Unlicensed driving can include driving with an expired, revoked, or suspended license, but in the case of adolescents, unlicensed driving is primarily a matter of driving before getting a license. Accordingly, we conceptualized unlicensed driving here as driving done by not-yet-licensed drivers, which has the added advantage of avoiding the conceptual complexity and resulting analytic complications of including the quite different phenomenon of losing a license through suspension or revocation due to misbehavior.

## Methods

### Study design

We examined data from motor vehicle crashes that involved two vehicles, in which the police reported that the actions of one of the drivers contributed to the occurrence of the crash (hereafter referred to as the “contributory driver”), while no action of the other driver was noted as contributing (“non-contributory driver”), and in which the non-contributory driver was 16 or 17 years old (16 is the minimum age to drive without adult supervision in NC). We assumed the non-contributory drivers played no role in the occurrence of these crashes other than by their mere presence, and thus that the distributions of various characteristics among non-contributory drivers in crashes approximate the corresponding distribution among all drivers on the road. Using this approach, we estimated the proportion of the total driving exposure of drivers aged 16–17 who were not yet licensed. The data analyzed were selected from all police-reported crashes that occurred in NC during 1991–2016.

### Data sources

#### Crash data

Crash data are reported by investigating officers to the NC Department of Transportation (NCDOT), which maintains the NC Crash Data File. Crash report data include extensive detail about the characteristics of each crash, each vehicle involved in the crash, and each crash-involved person including drivers, all passengers, and non-occupants (pedestrians, bicyclists). We examined crash data from 1991 through 2016 for the present study.

#### Driver license data

Driver licensing information is collected by the NCDOT whenever a license is issued, suspended, renewed, or any other license action is taken. For this study, we obtained licensing history data from 1980 through 2019, with information on the date and type of each license issued and all subsequent license actions. NCDOT grants permission to use both crash and license data for research purposes, so long as unique identifiers are not included in publications, re-disclosed, or used to contact individuals (stipulations detailed in the Federal Driver's Privacy Protection Act of 1994).

#### US census data

We geocoded drivers’ addresses as recorded in crash reports to the Census tract level and linked them with tract-level demographic information available from the US Census Bureau. The standard Census hierarchy includes (from largest to smallest): regions, divisions, states, counties, tracts, block groups, and blocks. The shapefiles used for geocoding changed between 1990 and 2010 in NC. Consequently, the same address might belong to a different block or block group with decennial updates. Addresses are much less likely to shift between larger area groupings like tracts. Therefore, we geocoded at the Census tract level because it generated a more stable solution. Although tracts comprise larger areas and are less precise than block groups and blocks, they provide a better indicator of neighborhood characteristics than county or zip code.

### Measures

#### Drivers’ licensing status

The NC driver license file maintains a complete history of an individual’s license actions, including all applications as well as licenses issued. This enabled us to determine whether crash-involved, non-contributory drivers had ever been licensed in NC by the time of their crash. Non-contributory crash-involved 16- and 17-year-old drivers for whom there was no record on any license issuance were coded as a not-yet-licensed driver.

#### Race/ethnicity

Driver race/ethnicity is included in NC crash and licensing data as a single variable with the following categories: White, Black, Indian (Native American), Hispanic, Asian, and Other. Drivers self-identify into only one of these categories, which precludes separate analyses of race and ethnicity. Prior to 2000, Hispanics and Asians were categorized as “Other,” and there were not enough crash-involved Native American drivers to analyze separately. Accordingly, analyses included only self-identified White and Black drivers. We examined whether drivers’ race may have influenced officers’ judgment of their contribution to the crash and found no differences between White and Black drivers by comparing the proportion in each group for which no driver action was indicated as contributing to the crash. These were virtually identical for White and Black 16–17-year-olds (32.1% vs. 31.8%).

#### Neighborhood income level

We measured the income level of drivers’ home neighborhoods using the median household income of the Census tract in which they resided. We considered Census tracts whose median income was in the lowest quartile for the state in a particular year to be low-income neighborhoods. Yearly income levels were adjusted to reflect 1999 US dollars.

To get a better sense of the validity of neighborhood median household income as an indicator of socioeconomic status, we scored drivers in the lowest income quartile in 2015 using the Area Deprivation Index (which was readily available only for 2013, 2014, and 2015; University of Wisconsin School of Medicine and Public Health 2015). Seventy-six percent of these drivers scored 7 or higher (with 1 reflecting the least disadvantaged neighborhoods and 10 the most disadvantaged). Accordingly, as previous studies have found (e.g., Anderson et al. 2018; Boone-Heinonen et al. 2010; Cubbin et al. 2005), median household income appears to be an efficient and reasonably valid indicator of neighborhood socioeconomic status.

### Creating the analysis file

We used the investigating officer’s assessment of whether a driver’s action contributed to the crash to identify non-contributory drivers in two-vehicle crashes in which only one driver was clearly contributory (in the QIE literature these are commonly referred to—somewhat confusingly—as “clean” crashes). (See the Appendix for a complete list of potential contributing driver actions recorded in the crash report.) We used this information instead of traffic citations to identify drivers who were involved in a crash merely because of their presence at the road location. Although citations are sometimes used for this purpose, driver actions are only one of several factors that influence whether officers issue citations (Curry et al. 2014; Tomczak, personal communication).

**Fig. 1:**
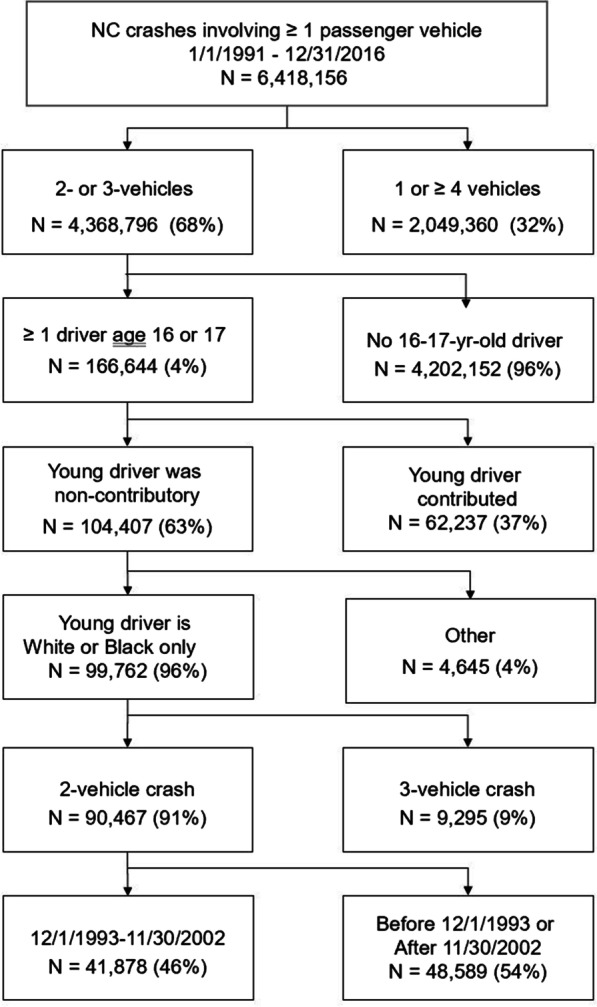
Identification of cases for analysis (included cases on left)

The first step in a QIE analysis is to validate the assumption that non-contributory drivers in two-vehicle crashes are representative of the general driving population. Following the standard procedure used to validate this assumption (Curry et al. 2016; Jiang & Lyles 2010; Zhao et al. 2019), we compared the characteristics of non-contributory drivers in two-vehicle crashes with those in three-vehicle crashes, as the latter group is assumed to be even more randomly selected from the road and thus more representative of the driving population. We identified 90,467 two-vehicle and 9,295 three-vehicle crashes from 1991 through 2016 involving only one contributory driver, and in which the non-contributory driver was aged 16 or 17. The comparisons revealed that the distribution of driver race, sex, and age in these two groups of crashes was highly similar (see Table 1). Therefore, it is reasonable to assume that the sample of non-contributory drivers was representative of all driving among 16- and 17-year-olds. The case identification process used to create the final analysis file is shown in Fig. 1.

**Table 1:** Characteristics of non-contributory 16- and 17-year-old North Carolina drivers in two- and three-vehicle crashes involving only one contributory driver

	Two-vehicle (*n* = 90,467)		Three-vehicle (*n* = 9,295)	
	*n*	%	*n*	%
Race				
White	76,848	85	8,141	88
Black	13,619	15	1,154	12
Sex				
Male	43,777	48	4,449	48
Female	46,690	52	4,846	52
Age				
16	39,005	43	3,865	42
17	51,462	57	5,430	58

Non-contributory means the crash investigator concluded the driver did nothing that contributed to the crash

### Analytic approach

The prevalence of unlicensed driving among adolescents in NC was estimated by identifying the proportion of non-contributory drivers in two-vehicle crashes who had never been licensed by the time of their crash. We then conducted logistic regression analyses to examine the likelihood a driver was unlicensed as a function of race (Black vs. White), neighborhood income level (lowest 25% vs. upper 75%), and licensing era (pre- vs. post-enactment of GDL). We examined the effect of GDL by comparing 5-year periods before (Dec 1, 1992–Nov 30, 1997) and after (Dec 1, 1997–Nov 30, 2002) it was implemented, which reduced the analytic sample to 41,878 cases. Further, we examined whether the introduction of GDL was associated with a differential change in unlicensed driving for drivers of different races and/or income levels. Separate logistic regression models were estimated for 16-year-olds and 17-year-olds. In keeping with sound epidemiological practice (Savitz 2013; Savitz & Wellnius 2016), as well as recent recommendations of the American Statistical Association (Wasserstein & Lazar 2016; Wasserstein, Schirm & Lazar 2019), we do not report p values; 95% confidence intervals are provided to help guide interpretation of parameter estimates.

## Results

### Prevalence of unlicensed driving among adolescents

**Fig. 2:**
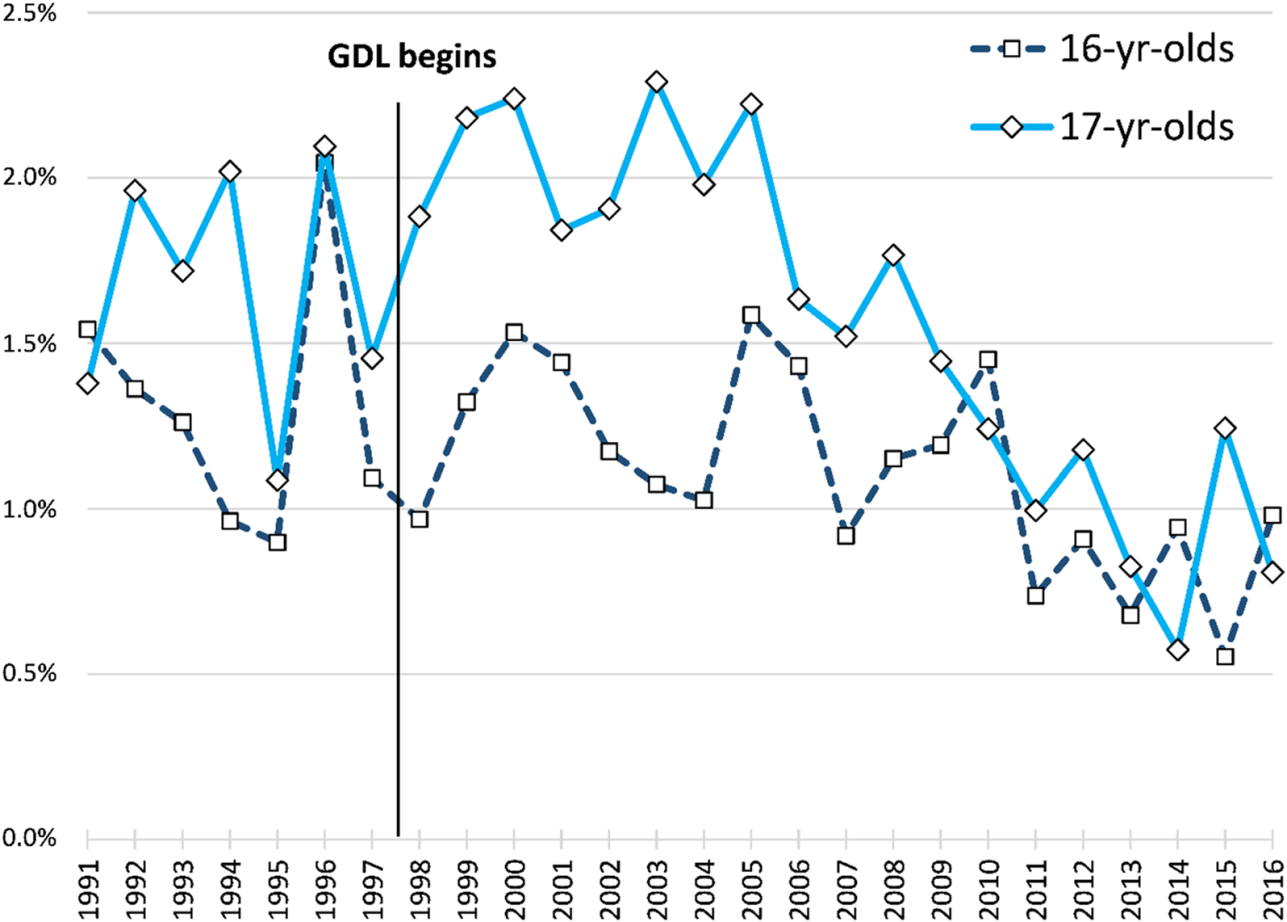
Estimated annual percent of driving done by not-yet-licensed North Carolina drivers, by age

During the 26 years for which data were available, the mean annual prevalence of unlicensed driving was 1.2% for 16-year-olds and 1.7% among 17-year-olds. As indicated in Fig. 2, yearly rates of unlicensed driving among those young enough to have possibly been influenced by GDL were somewhat erratic, occasionally differing by nearly a full percentage point from year to year. Nonetheless, the visual pattern suggests little or no change in unlicensed driving among either 16- or 17-year-olds for nearly a decade following implementation of GDL. By 2011, unlicensed driving had declined slightly among 16-year-olds and more notably among 17-year-olds.

### Unlicensed driving by race, neighborhood income level, and GDL

Table 2 shows the characteristics of the analytic sample that includes the 10-year period surrounding the adoption of GDL. Table 3 shows the number and percent of non-contributory drivers who were unlicensed for each subgroup of interest, along with crude and adjusted odds ratios (aORs), for the 5 years before and after GDL took effect. Overall, during this 10-year period unlicensed driving was quite rare among 16- (1.3%) and 17-year-olds (1.8%), with a combined rate of 1.6% (not shown in the table). The slightly higher prevalence of unlicensed driving among 17-year-olds compared to 16-year-olds was evident across most of the subgroups we examined. Black drivers were the exception to this pattern, with similar rates for 16- and 17-year-olds.

**Table 2:** Characteristics of final analytic sample of non-contributory 16- and 17-year-old North Carolina drivers in two-vehicle crashes during the 5-year period prior to and following adoption of GDL (*N* = 41,864)

Age			
	16	18,796	45%
	17	23,068	55%
Race			
	White	35,459	85%
	Black	6,405	15%
Neighborhood income			
	Upper 75%	32,077	77%
	Lower 25%	9,787	23%
Licensing era			
	Pre-GDL	22,031	53%
	Post-GDL	19,833	47%

Pre-GDL period is Dec 1, 1997, to Nov 30, 2002; post-GDL is Dec 1, 2002, to Nov 30, 2007

**Table 3:** Estimates of unlicensed driving among 16- and 17-year-old North Carolina drivers by licensing era, race and income

			16-year-olds					17-year-olds			
		Total	Unlicensed		Odds-ratio (CI)			Unlicensed		Odds-ratio (CI)	
			*n*	%	Crude	Adjusted	Total	*n*	%	Crude	Adjusted
*Licensing Era*											
	Pre-GDL	10,406	131	1.3%			11,634	193	1.7%		
	Post-GDL	8394	106	1.3%	1.00 (0.78; 1.3)	1.07 (0.83; 1.39)	11,444	230	2.0%	1.22 (1.00; 1.47)	1.27 (1.05; 1.54)
*Race*											
	White	16,496	163	1.0%			18,974	288	1.5%		
	Black	2304	74	3.2%	3.32 (2.52; 4.39)	2.96 (2.22; 3.94)	4104	135	3.3%	2.21 (1.79; 2.72)	1.79 (1.45; 2.22)
*Neighborhood income*											
	Upper 75%	14,569	149	1.0%			17,518	225	1.3%		
	Lower 25%	4231	88	2.1%	2.06 (1.58; 2.68)	1.71 (1.30; 2.24)	5560	198	3.6%	2.84 (2.34; 3.44)	2.56 (2.09; 3.12)

Pre-GDL period is Dec 1, 1997 to Nov 30, 2002; Post-GDL is Dec 1, 2002 to Nov 30, 2007. The first category listed is the reference group. aORs show adjustments for the other two variables (e.g., adjusting for GDL period and neighborhood income produces a slightly lower racial disparity in unlicensed driving among 17-year-olds (1.79 vs. 2.21)

Unlicensed driving was more common—though still relatively rare—among Black adolescents and those residing in the lowest-income neighborhoods. The combined rate of unlicensed driving among 16- and 17-year-old Black adolescents was 3.3% and 2.9% for adolescents in the lowest-income neighborhoods (combined age data not shown in table). Racial differences were slightly larger among 16-year-olds, whereas income level differences were somewhat larger among 17-year-olds. Controlling for race reduced income level effects slightly, and vice versa, as is indicated by differences between the crude and adjusted odds ratios in Table 3. The simple percentage differences as well as the odds ratios indicate that the prevalence of unlicensed driving following the adoption of GDL was unchanged among 16-year-olds and increased only slightly—if at all—among 17-year-olds.

Table 4 shows the results of the analysis to determine whether there were racial or income differences in unlicensed driving before and after GDL was enacted. The aORs shown for a race are adjusted for neighborhood and income, and those for income are adjusted for race. The race- and income-specific changes in unlicensed driving following the enactment of GDL mirror the overall findings, with little change (ORs mostly ranging from 0.88 to 1.08) among 16-year-olds and small increases among 17-year-olds (ORs ranging from 1.20 to 1.37). The only exception to this pattern is that unlicensed driving increased slightly among 16-year-old Black drivers (OR = 1.61) during the GDL era. Adjusting for race or income did not notably alter the basic unadjusted effects.

**Table 4:** Effects of GDL on unlicensed driving among 16- and 17-year-old North Carolina drivers, by race and income

			16-year-olds					17-year-olds			
		Total	Unlicensed		Odds ratio (CI)			Unlicensed		Odds ratio (CI)	
			*n*	%	Crude	Adjusted	Total	n	%	Crude	Adjusted
Licensing era and race											
	White, Pre-GDL	8994	94	1.0%			9409	130	1.4%		
	White, Post-GDL	7502	69	0.9%	0.88 (0.64; 1.2)	0.90 (0.66; 1.23)	9565	158	1.7%	1.20 (0.95; 1.51)	1.22 (0.96; 1.54)
	Black, Pre-GDL	1412	37	2.6%			2225	63	2.8%		
	Black, Post-GDL	892	37	4.1%	1.61 (1.01; 2.56)	1.61 (1.02; 2.57)	1879	72	3.8%	1.37 (0.97; 1.93)	1.40 (0.99; 1.98)
Licensing era and neighborhood income											
	Upper 75%, Pre-GDL	7895	78	1.0%			8720	100	1.1%		
	Upper 75%, Post-GDL	6674	71	1.1%	1.08 (0.78; 1.49)	1.13 (0.81; 1.56)	8798	125	1.4%	1.24 (0.95; 1.62)	1.26 (0.97; 1.64)
	Lowest 25%, Pre-GDL	2511	53	2.1%			2914	93	3.2%		
	Lowest 25%, Post-GDL	1720	35	2.0%	0.96 (0.63; 1.48)	0.99 (0.64; 1.53)	2646	105	4.0%	1.25 (0.94; 1.66)	1.29 (0.98; 1.71)

Pre-GDL period is Dec 1, 1997, to Nov 30, 2002; post-GDL is Dec 1, 2002, to Nov 30, 2007. White and upper-income neighborhood are the reference groups. aORs for race effects are adjusted for neighborhood income, aORs for income are adjusted for race

## Discussion

The goal of this study was to identify the prevalence of unlicensed driving among 16- and 17-year-olds in the NC driving population and to examine the associations of race, neighborhood income level, and GDL with unlicensed driving. Using a QIE approach, we found that for the 20-year period from 1991 through 2010, the prevalence of driving without having gotten a license was consistently between one and two percent among 16- and 17-year-olds. More recently (2011–2016), it declined, fluctuating around one percent yearly. Our detailed analyses focused on the 10-year period surrounding the date when GDL took effect (Dec 1, 1997). Unlicensed driving among 16-year-olds did not change after GDL was enacted and increased only slightly for 17-year-olds. Overall, our findings indicate that unlicensed driving is not nearly as common among adolescents as suggested by previous studies. It is possible, of course, that unlicensed driving in NC differs markedly from other states, but we know of no reason to think this would be the case.

By avoiding several shortcomings that routinely characterize self-report surveys, such as respondent self-selection, response set biases and other measurement difficulties, and sampling complications or inadequacies (Babbie 1990; Bailey & Wundersitz 2019), and by avoiding the inherently biased samples of drivers involved in crashes, the present analyses likely provide a closer approximation to the actual extent of unlicensed driving among adolescents. Though not without its limitations, the QIE approach has several advantages. Key strengths include the ability to study all 16- and 17-year-olds in a state who were actually driving, over a lengthy time period, using an objective indicator of whether they had a license. In contrast, previous surveys sampled groups of individuals who were eligible to obtain a license (but not necessarily driving) and willing to participate in a survey. Self-report studies typically measure the proportion of teens who say they have driven some arbitrarily selected amount (e.g., “regularly,” “an hour per week”), or the kinds of trips driven without a license. Most importantly, because all these measures lack an adequate denominator, they simply tell us what proportion of a population has engaged in some version of unlicensed driving. They are unable to address the more pertinent question of what proportion of all adolescent driving is done by those who are not yet licensed. In principle, this is captured by the sampling approach QIE analysis embodies. Unlicensed drivers are “sampled” (by contributory drivers) in proportion to their prevalence in the total mileage driven in a jurisdiction, rather than their simple proportion among all drivers or among an age-group.

One of the key motivations for this study was to examine whether unlicensed driving was more common among adolescents in minority and low-income families. Previous research has indicated that being Black and living in poor neighborhoods is associated with driving without having gotten a license. Our results replicated those findings; however, the difference in unlicensed driving we found in NC between Black and White drivers is about half what Elliot et al. (2008) reported for a nationally representative sample and minuscule in comparison with the differences reported by Heck et al. (2008).

Dozens of studies indicate that well-designed GDL programs are highly effective in reducing adolescent driver crashes (Williams 2017). Nonetheless, there has been concern that logistic barriers to complying with GDL requirements among minority and economically disadvantaged teenagers may result in more unlicensed driving (Shults et al. 2016). Although such concerns seem reasonable, our findings suggest that the practical effect of GDL on unlicensed driving among Black and lower-income adolescents was extremely small. Although proportionate increases ranging from 20 to 37% may seem noteworthy, it is important to keep in mind that these are increases in quite low base rates. For example, the 25% increase in unlicensed driving after GDL took effect among 17-year-olds living in low-income neighborhoods represents an absolute change of only 0.8% (from 3.2 to 4.0%).

Although the QIE approach offers many advantages over previous studies, this study does have a few limitations. First, to the extent that not-yet-licensed adolescent drivers were more likely than those with a license to leave the scene of a crash, our findings would underestimate the prevalence of unlicensed driving. Another question is whether not-yet-licensed drivers are more likely than licensed drivers to contribute to crashes. Although drivers with revoked or suspended licenses are more likely to crash (Brar 2014), virtually nothing is known about the actions of young crash-involved drivers who have not yet gotten a license. Having lost a license for repeated misbehavior and not yet having gotten a license are quite different phenomena, so we cannot assume findings about the former inform the latter. A final consideration is a possibility that adolescent drivers’ income level or lack of a license may have biased investigating officers’ judgment of whether the driver did something that contributed to the crash. Our analyses found no officer bias based on race; however, if officers were influenced by the licensing status of the adolescent driver, our analyses could underestimate the prevalence of unlicensed driving.

## Conclusions

Using a unique analytic approach, this study found that unlicensed driving among adolescents is substantially less common than suggested by self-report studies and analyses of the license status of crash-involved drivers. Young Black drivers and individuals living in lower-income neighborhoods are somewhat more likely than young White drivers and less disadvantaged adolescents to drive before obtaining a license, but the rates of unlicensed driving among these groups are also quite low. Unlicensed driving increased slightly for 17-year-olds following implementation of GDL but returned to previous levels within a few years.

## Data Availability

The datasets generated and analyzed during the current study are not publicly available due to a data use agreement with NCDOT.

## Appendix: List of possible driver contributing circumstances on the NC crash report form

**Table:**

0. No contributing circumstances indicated
1. Disregarded yield sign
2. Disregarded stop sign
3. Disregarded other traffic signs
4. Disregarded traffic signals
5. Disregarded road markings
6. Exceeded authorized speed limit
7. Exceeded safe speed for conditions
8. Failure to reduce speed
9. Improper turn
10. Right turn on red
11. Crossed centerline/going wrong way
12. Improper lane change
13. Use of improper lane
14. Overcorrected/oversteered
15. Passed stopped school bus
16. Passed on hill
17. Passed on curve
18. Other improper passing
19. Failed to yield right of way
20. Inattention
21. Improper backing
22. Improper parking
23. Driver distracted
24. Improper or no signal
25. Followed too closely
26. Operated vehicle in erratic, reckless, careless, negligent, or aggressive manner
27. Swerved or avoided due to wind, slippery surface, vehicle, object, non-motorist
28. Visibility obstructed
29. Operated defective equipment
30. Alcohol use
31. Drug use
32. Other* (Write in Narrative)
33. Unable to determine
34. Unknown

## Abbreviations

GDL: Graduated driver licensing
QIE: Quasi-induced exposure
NCDOT: North Carolina Department of Transportation
OR: Odds ratio
aOR: Adjusted odds ratio

## Footnotes

1 Vaca et al. did report that the minimum age at which licensing can begin was weakly associated with licensure occurring later than the minimum allowable age. However, licensing age is separate issue and not an element of the GDL process, though it is often mistakenly thought to be (the term GDL is often conflated with all elements of a state’s young driver licensing system). The full three-step GDL process can be implemented for young beginning drivers whether the minimum licensing age is 14, 17 or anything in between.
